# Her-2 Positive Gastric Cancer Presented with Thrombocytopenia and Skin Involvement: A Case Report

**DOI:** 10.1155/2014/194636

**Published:** 2014-06-23

**Authors:** Deniz Arslan, Mukremin Uysal, Ali Murat Tatlı, Seyda Gunduz, Sema Sezgin Goksu, Cumhur İbrahim Başsorgun, Hasan Senol Coskun, Hakan Bozcuk, Burhan Savaş

**Affiliations:** ^1^Department of Medical Oncology, Faculty of Medicine, Akdeniz University, 0700 Antalya, Turkey; ^2^Department of Medical Oncology, Faculty of Medicine, Afyon Kocatepe University, Izmir Karayolu 8.km, 03200 Afyon, Turkey

## Abstract

Gastric cancer is the 5th most frequent cancer around the world and the 3rd most frequent reason of deaths due to cancer. Every year, about 1 million new cases are taking place, with varying geographical distribution. Gastric cancer is often metastatic to liver, lungs, and bones in hematogenous way, to peripheral lymph nodes in lymphogenous way, and to peripheral tissues in adjacency way, yet bone marrow (BM) and cutaneous metastasis are quite seldom. Pancytopenia is a more frequent finding identified in BM metastasis of solid organ cancers, and isolated thrombocytopenia is less often. The human epidermal growth factor 2 (HER-2) is positive in gastric cancer at a rate of 7–34%. Here, we have presented our HER-2 positive gastric cancer incident which presented with BM and cutaneous metastasis, and has no 18F-fluoro-2-deoxi-D-glucose (FDG) involvement except bone metastases.

## 1. Introduction

Although gastric cancer incidences have been reduced as of the second half of the 20th century, it is still the 5th most frequent cancer around the world and the 3rd most frequent reason of deaths due to cancer. Every year, about 1 million new cases are taking place. This frequency presents an extremely different geographical distribution based on* Helicobacter pylori* infection, economic conditions, life style, and diet [1–4].

While gastric cancer is often metastatic to liver, lungs, and bones in hematogenous way, to peripheral lymph nodes in lymphogenous way, and to peripheral tissues in adjacency way, bone marrow (BM) and cutaneous metastasis are quite rare compared to other internal organ tumors and are detected within the late period of the disease [5–8]. Pancytopenia is a more frequently detected outcome in BM metastases of solid organ cancers; thrombocytopenia is seen less often [9].

Despite many developments in diagnosis and treatment, the average lifetime in advanced stage and recurrent gastric cancer is 6 to 9 months [6]. For the medical oncologists, treatment of the metastatic gastric cancer is even more difficult in cases where BM and cutaneous metastases are detected which are particularly seen in the advanced periods of the disease [6, 9]. Human epidermal growth factor-2 (HER-2) is positive in gastric cancer at a rate of 7–34% [10]. In recent years, by utilizing the treatments aiming at HER-2, better results were taken in gastric cancer incidents compared to the traditional treatments [11].

Here, we have presented our HER-2 positive gastric cancer incident which presented with BM and skin metastases and has no 18F-fluoro-2-deoxi-D-glucose (FDG) involvement except bone metastases in PET.

## 2. Case Report

**Figure 1:**
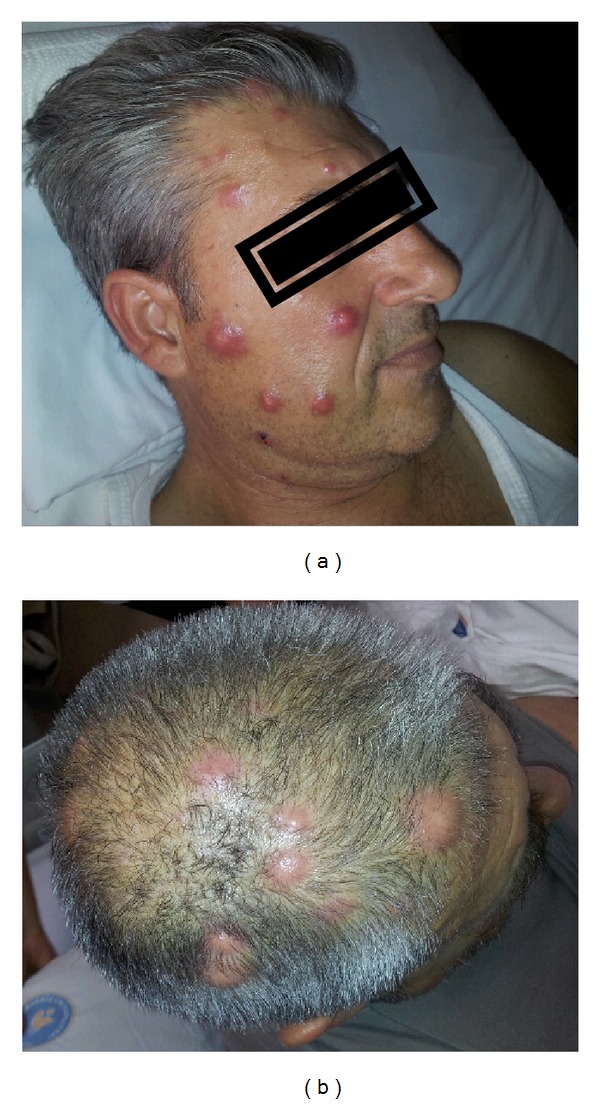
Large number of bulky, hyperemic nodules on the (a) face and (b) scalp.

**Figure 2:**
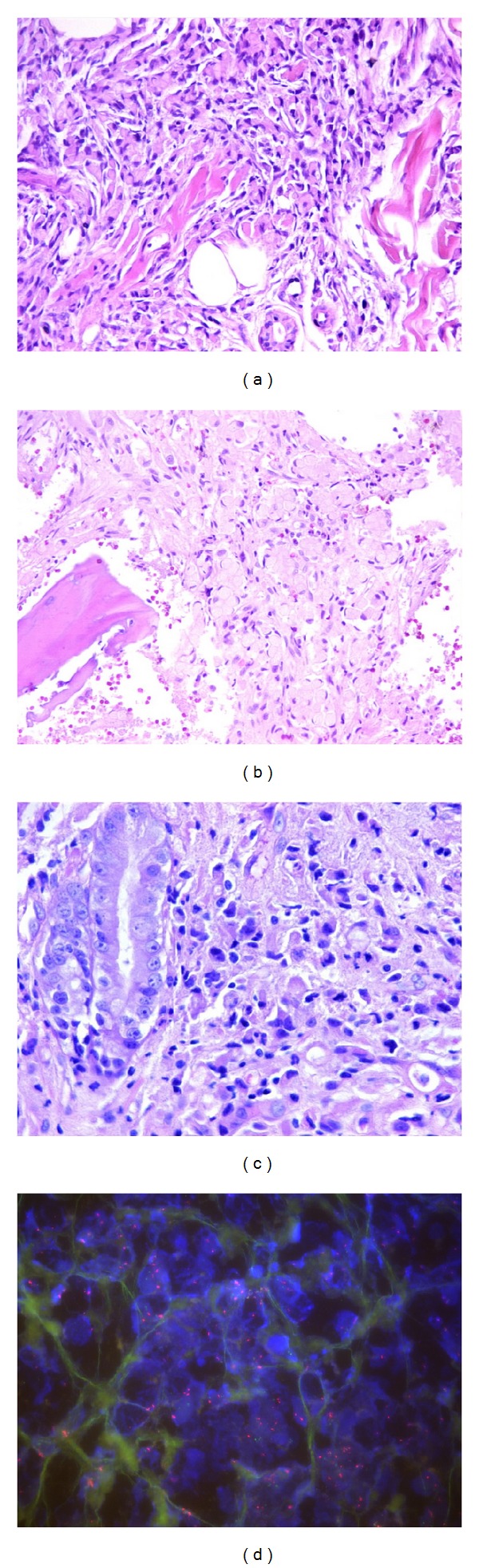
(a) Invasive and signet ring celled tumor infiltration on the dermis between the collagen fibers (HEX200). (b) In the paratrabecular area between the bone trabeculas, fibrosis and signet ring celled tumor are being observed (HEX100). (c) Signet ring cell-like tumor cells with large hyperchromatic nucleus in the stomach mucosa (HEX200). (d) HER-2-neu positiveness determined by the FISH method in the nucleus.

**Figure 3:**
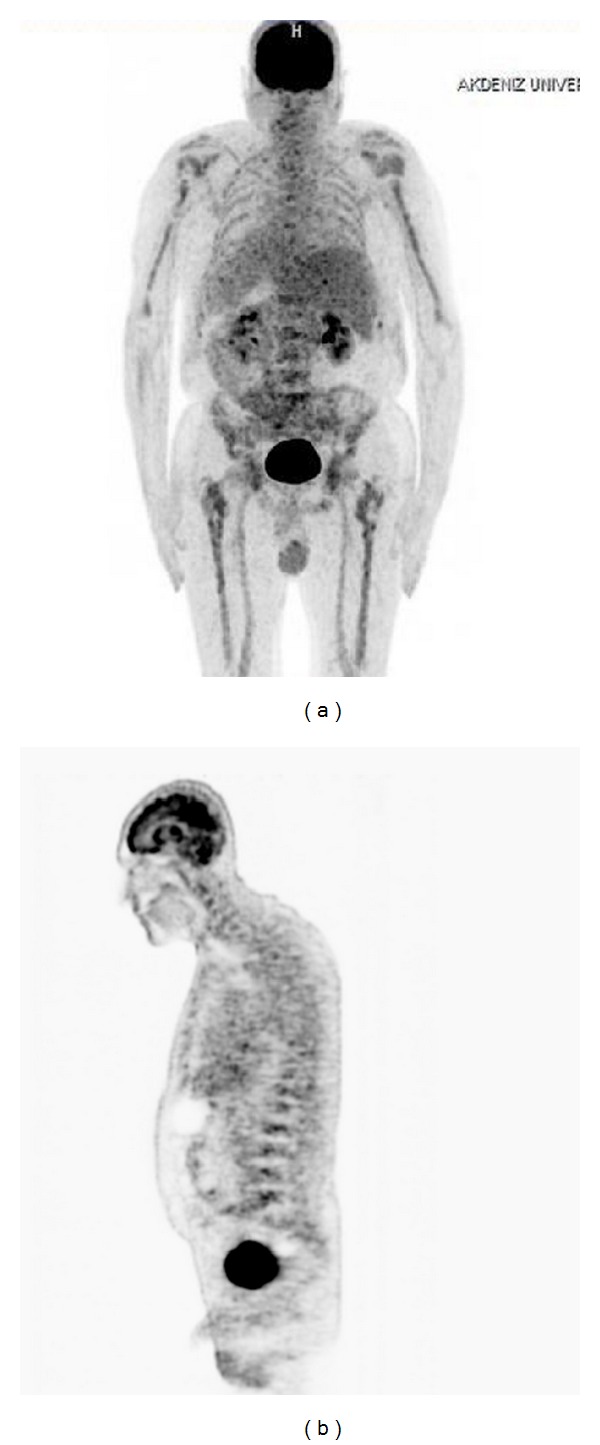
This figure shows no 18F-fluoro-2-deoxi-D-glucose (FDG) involvement except bone metastases in PET ((a) coronal section and (b) sagittal section).

Fifty-two-year-old male patient has presented with recently increased complaints which started 1 year ago such as pain in his waist, back, and hips, and 2 months prior to his hospital visit maculopapular and erythematous lesions (Figure 1) appeared on his arm. Upon the physical examination of the patient who has lost 8 kilograms within the last 5 months and was under warfarin treatment due to diagnosis of pulmonary thromboembolism by August 2011, his thrombocyte was detected as 36000 u/L. In all the bone structures included in computed tomography (CT) and imaging, no pathological outcome was found other than lytic lesions and the lesions were assessed as solid organ metastasis or multiple myeloma outcome. No monoclonal band was detected in serum and urine immunoelectrophoresis. Diagnostic excisional biopsy was applied to 1.5 × 1 cm nodule on the right forearm. In its pathology, tumoral tissue which has the feature of a signet ring cell (Figure 2(a)) was seen at some areas of malign epithelial tumor metastasis that was positively stained with Pan CK applied with immunohistochemical (IHC) method and negatively stained with CD20, CD3, LCA, S-100, and CD68 and biopsies of skin metastases were negatively stained with Her-2. In his whole body scintigraphy especially of the axial and appendicular skeletal system, diffuse and focal activity involvement was observed. According to the BM biopsy performed due to thrombocytopenia, Pan CK, mucin, PAS, d-PAS, and Her-2 were reported as positive, and hypercellular BM was observed including malign epithelial tumor cells with a signet ring cell view (Figure 2(b)). In the axial and appendicular skeletal system, lytic/sclerotic and heterogeneous diffuse/local hypermetabolic changes on FDG PET were detected (Figures 3(a) and 3(b)). In the upper gastrointestinal system endoscopy, a lesion with polypoid view was observed which started from the small curvature and extended near the pylorus and had a widespread eroded and ulcerous area on itself. As a result of the examination of the pathology of the multiple biopsies taken from the mass lesion by IHC method, it was reported as signet ring cellular carcinoma which was diffuse strong positive with CK7, showed mucicarmine and intracytoplasmic staining with PAS, and was 40% 3+ with Her-2 (Figure 2(c)). The positiveness of Her-2 was verified by the fluorescent in situ hybridization (FİSH) method (HER-2-neu/CEN 17 = 116/25 = 4.6) (Figure 2(d)). Palliative radiotherapy (3000 cGy) was applied to the lytic areas with high fracture risk in the lumbar vertebra and pelvic area determined by magnetic resonance (MR).

**Figure 4:**
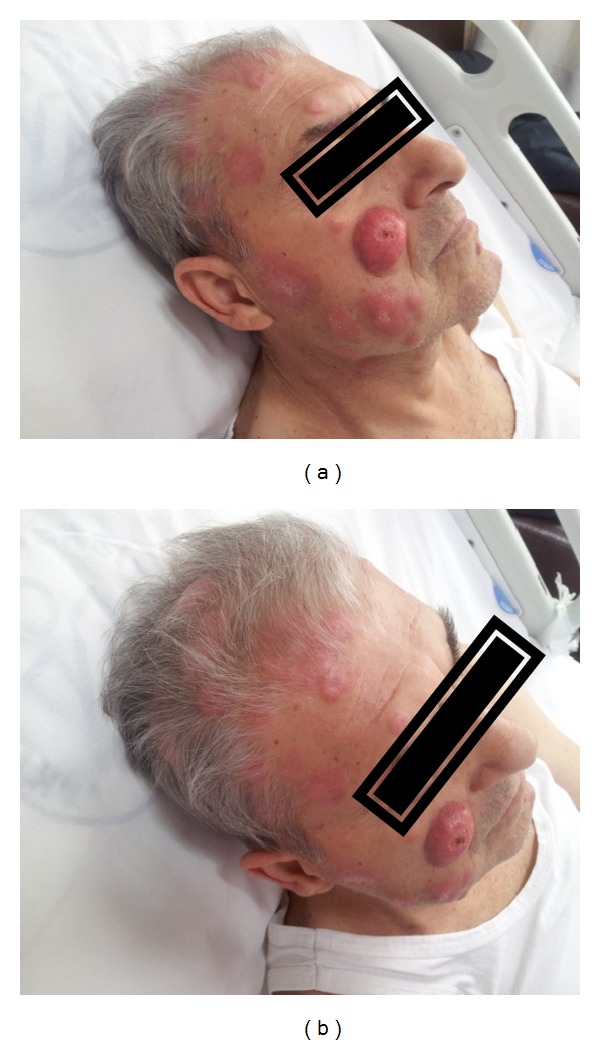
Progression of Her-2 negative skin metastases after 6th cycle of chemotherapy. (a) Face and (b) scalp.

Thrombocyte suspension support was applied to the patient and a combination chemotherapy including cisplatin, 5-fluorouracil, and trastuzumab (CFT) was started. Six cycles of CFT chemotherapy were applied. Platelet count was increased to the normal levels after the first cycle of treatment and there was no need for additional platelet infusion. Performance status was improved to the normal limits. Complete response for bone metastasis was obtained on PET-CT imaging. However, Her-2 negative skin metastases were progressed after 6 cycles of chemotherapy (Figure 4). CFT chemotherapy protocol was stopped due to the progression of disease after 6th month of initial treatment.

## 3. Discussion

In solid organ malignancies, BM metastasis can be observed in breast, prostate, and gastric cancers [9]. Recent studies reveal that there is resistance to traditional treatments in gastric cancer presented with bone marrow metastasis and the patient rapidly deteriorates [6, 9]. It is reported that the most frequent symptoms in gastric cancer cases with BM metastases are bone pain (43.6%), active bleeding (20.6%), dyspnea (12.8%), stomach pain (5.1%), and fatigue (5.1%) [12]. Even though pancytopenia is frequently observed as a laboratory finding, thrombocytopenia is also observed in rare instances. Median survival is 20 days under the best supportive care and 67 days with chemotherapy in this patient group [9]. In another study, the median overall survival after detection of BM metastases for the cohort of patients was 37 days (95% confidence interval: 12.5 to 61.5 days). The median overall survival after detection of BM involvement was 11 days in the best supportive care group (range: 2 to 34 days) and 121 days (range: 3 to 383 days) in the palliative chemotherapy group (*P* < 0.001) [6]. Therefore, when BM metastasis is present, palliative chemotherapy is the preferred treatment modality.

Gastric cancer constitutes about %2.2 of all skin metastasis cases [13]. Incidence of skin metastasis in all gastric cancers is 0.8% and is considered very rare. Skin metastases frequently affect the head, neck, breast, and abdominal wall. Skin metastases in gastric cancer are generally multiple nodules and sometimes cellulites or erysipelas-like lesions [5, 7, 13–15]. For the diagnosis of skin metastasis, histopathological features of biopsies conducted on lesions are important and generally show similarity with primary tumor [7]. Determination of skin metastasis in gastric cancer is associated with poor prognosis, similar to BM metastasis, and the mean survival after diagnosis is 11.4 weeks [7]. Although palliative chemotherapy and radiotherapy have been applied in many patients with skin metastasis, unsuccessful results have been achieved [7]. In our case, bone marrow and skin metastases were present. Trastuzumab treatment would have effectively prolonged his survival unless his skin metastasis was not Her-2 positive. So, this situation neutralized the advantages of the treatment targeted to Her-2.

Computerized tomography (CT) is the preferred method in the preoperative examination and staging of the stomach cancer. CT is also preferred to determine the tumor recurrence and the response to the treatment. Although PET is useful diagnostic tool in clinical oncology, its use in the assessment of stomach cancer is limited [16]. Besides, PET was found to be impractical in the diagnosis of stomach cancer when compared to its use in the diagnoses of other solid tumors according to a number of studies [17, 18]. When PET use is compared to that of CT in diagnosing stomach cancer, it is slightly better in determining the LN positiveness. Thus, PET is effective in determining resectability and distant metastasis but its use in locoregional staging is limited [16, 17]. In our case report, higher FDG uptake was not present in the stomach that was the primary tumor region. Higher FDG uptake was seen in skeletal system. Accordingly, PET-CT reveals false negative results in tumors with unknown primary.

The prognosis of metastatic stomach cancer is quite poor despite the developments in diagnosis and treatment modalities [19]. It is reported that HER-2 positiveness in stomach cancer varies between 7% and 34% [10]. In the ToGA (trastuzumab in the treatment of stomach cancer) study, HER-2 positiveness was determined as 22.1%. It was shown that the use of combination chemotherapy was efficient in the patient group having metastatic stomach cancer where monoclonal antibodies targeting HER-2 were also added [10, 16]. In our case report, Her-2 was positively stained in stomach and bone marrow and negatively stained in skin metastasis. As a result, stomach and bone marrow tumor were improved after the treatment with chemotherapy including trastuzumab; on the other hand, skin metastases progressed.

As a result, BM and skin involvement in stomach cancer is rarely seen and it is related to very poor prognosis. When a patient presents with thrombocytopenia, a solid organ tumor metastasis to BM should be considered in the definitive diagnosis. Because PET is insufficient for determining and staging of the stomach cancer, if necessary, additional examinations and interventions should be attempted. For the patients having BM or skin metastases due to stomach cancer, their treatment strategies should include systemic chemotherapy regimen in which target-driven treatments are combined with supportive treatment according to the status of the patient.
